# Wilson’s Disease Masquerading as Juvenile Idiopathic Arthritis in an Adolescent

**DOI:** 10.7759/cureus.9160

**Published:** 2020-07-12

**Authors:** Rajeev Goyal, Swathi Chacham, Jagdish P Goyal, Madhuradhar Chegondi

**Affiliations:** 1 Biochemistry, Lady Hardinge Medical College, New Delhi, IND; 2 Pediatrics, All India Institute of Medical Sciences, Rishikesh, IND; 3 Pediatrics, All India Institute of Medical Sciences, Jodhpur, IND; 4 Pediatrics, University of Iowa Stead Family Children's Hospital, Iowa City, USA

**Keywords:** wilson’s disease, juvenile idiopathic arthritis, children

## Abstract

Wilson’s disease (WD) is a rare autosomal-recessive inborn error of copper metabolism characterized by the toxic accumulation of copper in liver, brain, cornea, and other tissues. It has a variable clinical presentation. Musculoskeletal presentations are very unusual. We report a 17-year-old male who presented to us with juvenile idiopathic arthritis (JIA), which later proved to be a case of WD.

## Introduction

Wilson's disease (WD) is a metabolic disorder, which has autosomal-recessive inheritance, that involves a defect of copper transport by the hepatic lysosomes. The symptoms are nonspecific, and the disease generally presents itself as a hepatic disease or a progressive neurological disorder. It is often misdiagnosed at the initial presentation, which leads to delay in diagnosis and management [1]. We report an adolescent male, initially diagnosed with juvenile idiopathic arthritis (JIA), who was ultimately diagnosed with WD.

## Case presentation

A 17-year-old male presented to a general practitioner with a three-month history of bilateral ankle and knee pain and swelling. In addition, there was a history of morning stiffness. There was no history of trauma, fever, skin rash, oral ulcer, or alopecia. His exam at a community clinic was found to be normal except for the ankle and knee joint swelling and pain on movement. He was diagnosed as oligo-articular JIA and started on naproxen, iron, and calcium. Despite receiving this therapy for three months, there was no resolution of joint symptoms. This adolescent boy was subsequently referred to our clinic.

**Figure 1:**
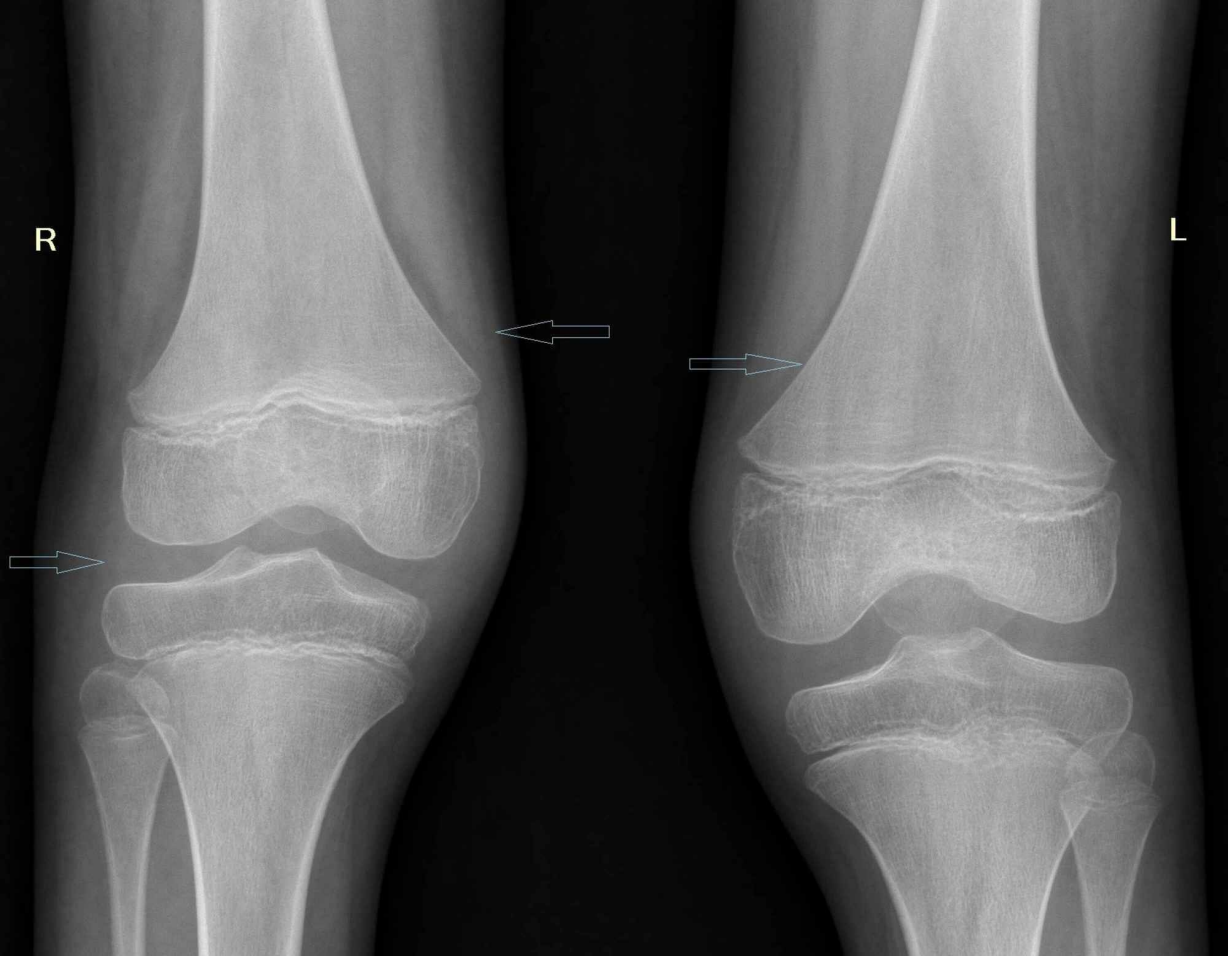
Bilateral knee joint X-ray showing soft tissue swelling with mild osteopenia

Exam at our clinic demonstrated normal vital signs, scleral icterus, conjunctival xerosis, Bitot's spots, hepatomegaly (2 cm below the right costal margin) without splenomegaly or ascites. The rest of his examination was unremarkable. His laboratory workup revealed a normal complete blood count (CBC) with hemoglobin of 12.8 gm/dL, total leukocyte count of 6.3 × 103 / μL, platelet count of 202 × 103/ µL inflammatory markers elevated, C-reactive protein (CRP) 16 mg/dL and erythrocyte sediment rate (ESR) 40 mm/h. His liver function tests showed an elevated total bilirubin, 2.8 mg/dL (normal value 1 mg/dL) , alanine transaminase (ALT) 110 IU/L (normal level 7-56 IU/L), aspartate transaminase (AST) 171 IU/L (normal levels 5-40 IU/L), elevated alkaline phosphatase 460 IU/L (normal 74-390 IU/L), total protein 6 gm/dL, low albumin 2.7 gm/dL (normal levels 3.4-5.4 g/dL), globulin 3.7 gm/dL. His renal functions were normal, with blood urea nitrogen 20 mg/dL, creatinine 0.78 mg/dL. His human leukocyte antigen B27 (HLA-B27) assay was negative. Bilateral knee joint X-ray showed soft tissue swelling with mild osteopenia (Figure 1). 

**Figure 2:**
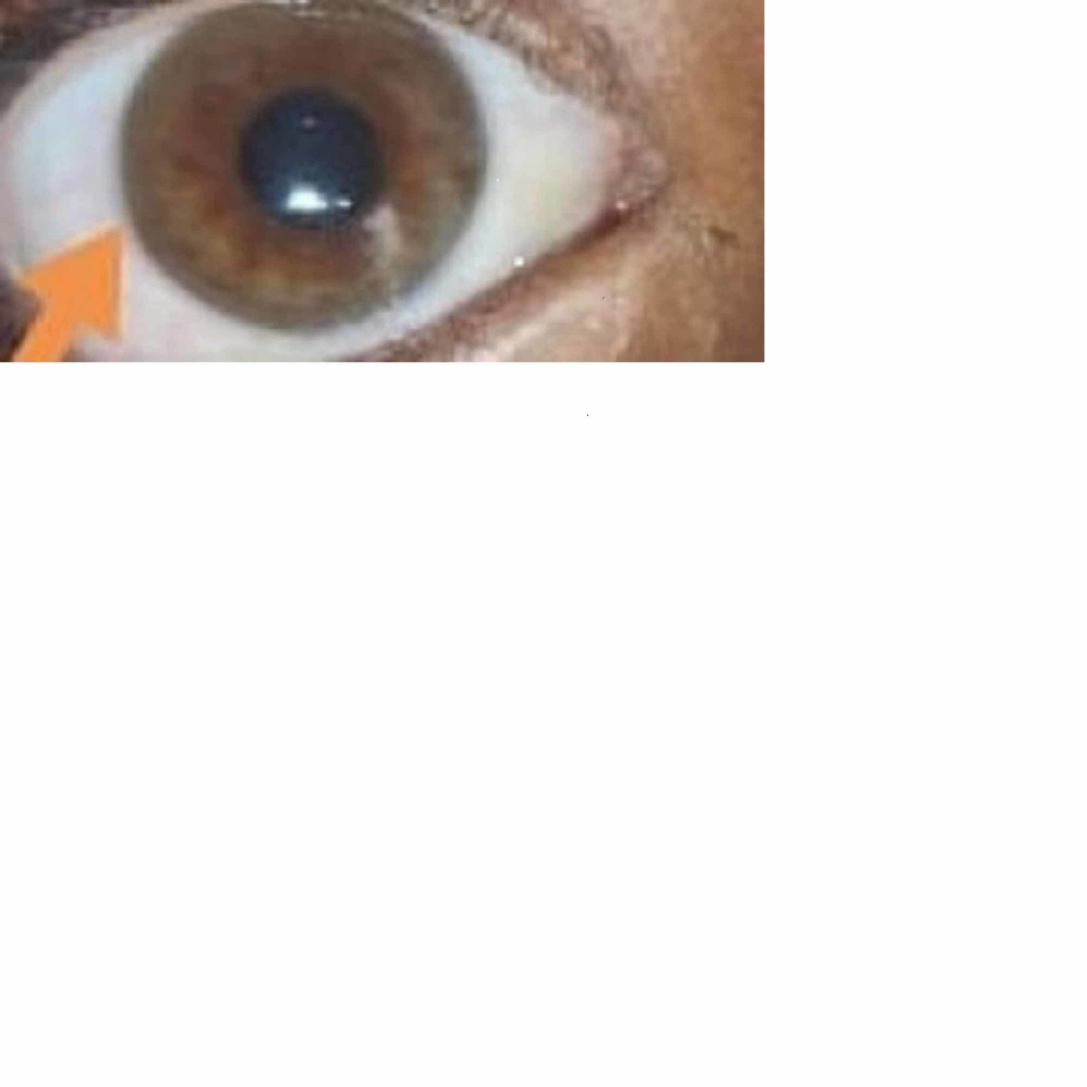
Slit-lamp examination of the eye showing the Kayser-Fleischer ring

Given the transaminitis and hyperbilirubinemia, the patient was evaluated for chronic liver disease. Viral markers were found to be negative. Evaluation of autoimmune hepatitis (antinuclear antibody and anti-LKM) was also negative. Additional evaluation demonstrated a low serum ceruloplasmin level, 7.5 mg/dL (normal level 20-40 mg/dL), and an elevated 24-hour urinary copper level, 113.17 µg/24 h (normal level <40 µg). Slit-lamp examination demonstrated Kayser-Fleischer (KF) rings (Figure 2).

No neuroimaging is done, as he did not have any neurological symptoms. The boy was diagnosed with WD, and therapy with D-penicillamine and zinc acetate was commenced. He was discharged home to follow-up after one month. He was also started on vitamin A supplementation for Bitot’s spot and conjunctival xerosis. At the follow-up visit, the patient showed significant clinical improvement with the resolution of jaundice, joint pain, and swelling. 

## Discussion

We report an adolescent boy, initially diagnosed with JIA, who was ultimately diagnosed with WD. WD is a rare genetic disorder with an estimated prevalence of 1 in 30,000 [1]. A higher prevalence of 1 in 1,500 to 3,000 is noted in East Asian countries [2]. There is defective copper transport by hepatic lysosomes due to impaired function of metal transporting P-type adenosine tri-phosphatase (ATPase), encoded by the ATP7B gene on chromosome 13 [2]. Decreased biliary excretion of copper causes its accumulation in hepatocytes. As copper does not bind to ceruloplasmin, it is secreted without copper, producing apoceruloplasmin [2,3].

The clinical presentation of WD is highly variable, which causes a delay in diagnosis. It has been stated that no two patients of WD may have similar clinical characteristics even among siblings, though hepatic and neurological manifestations are usually early presenting features [4]. The hepatic presentation can vary from asymptomatic elevated liver chemistries to acute hepatitis, and cirrhosis. Neurological manifestations present as WD progresses into the second or third decade of life, which include dysphagia, dysarthria, seizures, dystonia, ataxia, flapping, or rest tremor, autonomic and behavioral disturbances [5]. In contrast to hepatic disease, the K-F ring is always noted in individuals with neurological involvement secondary to WD [4]. Interestingly, although our patient did not have neurological symptoms, he had a K-F ring.

Articular presentation in children with WD is rare and is usually in the absence of typical hepatic or neurological symptoms [6]. Isolated articular symptoms in WD were reported previously as few case reports, similar to our index case [7,8]. Mono or polyarthritis with knee joint involvement is common [6]. The underlying mechanism for joint involvement is unknown; however, recurrent microtrauma to the joints and direct copper toxicity on joint tissue are postulated as possible mechanisms [9]. The radiological findings include degenerative changes of the affected joints, osteoporosis, osteomalacia, osteochondritis, and fractures [9]. The typical biochemical features of WD include low serum ceruloplasmin, less than 20 mg/dL, low serum copper level, and increased urinary copper levels [5]. If the diagnosis is inconclusive, mutation analysis for ATP7B gene and liver copper content measurement may be necessary [5]. The MRI of the brain shows bilateral hyperintensities of basal ganglia and thalamus [2].

Three clinical practice guidelines are available till date to diagnose WD [10]: the American Association for the Study of Liver Diseases (AASLD), the European Association for the Study of the Liver (EASL) and the European Society for Pediatric Gastroenterology, Hepatology and Nutrition (ESPGHAN) [5]. All three guidelines differ in their approach to diagnose WD. While AASLD guideline use algorithmic approach to diagnose WD, the EASL and ESPGHAN guideline relies on Leipzig score to establish the diagnosis. Though we did not perform a liver biopsy and genetic testing, however, our case fulfilled the criteria for the diagnosis of WD as per the above guideline. If the KF ring is present, serum ceruloplasmin is <20 mg/dL, and 24 urine copper excretion is >40 µg, then the diagnosis of WD is established as per the AASLD guideline. Our patient also had Leipzig score >4, suggesting the diagnosis of WD as per the EASL and ESPGHAN guidelines.

D-penicillamine is the initial chelating agent of choice in WD, and treatment is lifelong [2,5]. Other copper chelating agents that may be used are trientine and tetrathiomolybdate [2]. While the chelating agents promote copper excretion, zinc helps to prevent the intestinal absorption of copper [2,5]. Chelating agents are effective in most patients with WD. Both D-penicillamine and trientine have a similar outcome. Although the rate of adverse effects is less with trientine, it is expensive [11].

In our index case, the initial diagnosis of JIA was based on clinical presentation and elevated ESR and CRP. The absence of hepatic or neurologic involvement in the setting of arthritis, is a diagnostic challenge for WD [12]. The presence of scleral icterus and transaminitis during his subsequent visit to our hospital, and the further workup for hepatitis and led to the diagnosis of WD. Following therapy with D-penicillamine and zinc acetate, the adolescent showed a significant improvement in his joint symptoms and liver function.

## Conclusions

WD typically presents with hepatic or neurological features in children. However, the presentation can be variable and rarely as isolated joint involvement. One should always consider genetic or metabolic diagnoses such as WD if articular symptoms are suggestive of JIA and not responding to standard treatment. Arthritis associated with WD promptly responds to chelation therapy by D-penicillamine.
